# Tendency of microbial adhesion to denture base resins: a systematic review

**DOI:** 10.3389/froh.2024.1375186

**Published:** 2024-05-16

**Authors:** Firas K. Alqarawi, Mohammed M. Gad

**Affiliations:** Department of Substitutive Dental Sciences, College of Dentistry, Imam Abdulrahman Bin Faisal University, Dammam, Saudi Arabia

**Keywords:** 3D printing, CAD-CAM milled, microbial adhesion, complete dentures, digital dentures

## Abstract

**Objectives:**

Digital denture fabrication became an alternative method to conventional denture fabrication. However reviewing the antimicrobial performance of newly introduced digital fabrication methods in comparison to the conventional method is neglected. Aim of study: this review was to compare the antiadherence properties of various CAD-CAM subtractive (milled), additive (3D printed) conventional denture base resins. In order to answer the developed PICO question: “Does CAD-CAM milled and 3D printed denture base resins have microbiological antiadherence properties over the conventional ones?” We included comparative studies on digitally fabricated Denture base resins with conventionally fabricated one in term of microbial adhesion.

**Methods:**

All *in vitro* studies investigated the microbial adherence to CAD-CAM milled and 3D printed denture base resins in comparison to conventional were searched in the PubMed, Web of Sciences, and Scopus databases up to December 2023.

**Results:**

Fifteen studies have been investigated the microbial adhesion to milled and 3D printed denture base resins. CAD-CAM milled resins significantly decreased the microbial adhesion when compared with the conventional resins and 3D printed resins, while the later showed a high tendency for microbial adhesion. The addition of antifungal agents to 3D printed resins significantly reduced *C. albicans* adhesion. In terms of 3D printing parameters, printing orientation affected adherence while printing technology had no effect on microbial adhesion.

**Conclusion:**

Denture base materials and fabrication methods significantly affect the microbial adhesion. CAD-CAM milled denture base resins demonstrated low microbial adhesion. 3D-printed resins showed high tendency for *C. albicans* adhesion. The antiadherent properties of 3D-printed resins can be improved by incorporating antifungal agents or changing the printing parameters, but further investigations are required to validate these modifications.

## 1 Introduction

The most common clinical problem associated with patients wearing complete dentures is denture stomatitis (DS). This infection is primarily caused by *Candida albicans* adhesion to the denture base surface (1). Surface properties are considered the most important factor in *C. albicans* adhesion and colonization, along with other factors such as poor oral hygiene and ill-fitting dentures (2). It was reported that DS occurrence rate is about 30%–75% of denture wearers and high recurrence rate even with antifungal treatment (3). This situation increased as the surface properties change (increasing Ra and contact angle and decreasing hardness) where rougher surfaces act as a nest and become an adequate environment for microbial adhesion and colonization (2). The surface properties of denture base resins affected by the fabrication method, and CAD-CAM milled denture base resins had superior surface properties (4–6). Therefore, denture base resins with smooth surfaces that are less appealing to microbial adhesion contributed to denture longevity when combined with healthy denture foundation tissue.

For digital denture fabrication, the use of computer-aided-design-computer-aided-manufacture (CAD-CAM) methods is becoming more popular. This is due to many advantages over conventional method such as reducing the number of appointments, laboratory time required for prostheses fabrication, reducing laboratory errors, and the ability to store data for future fabrication (7–9). CAD-CAM fabricated prostheses demonstrated better adaption than conventionally-fabricated ones (10) in addition to their superior physical properties (11). CAD-CAM denture fabrication includes two methods; milling denture from prepolymerized Polymethylmethacrylate (PMMA) acrylic discs that polymerized under high pressure/temperature (subtractive method, SM) and building the denture in layers using photopolymerized resins (additive method, AM) also known as three-dimensionally (3D) printed denture base resins (12, 13). SM is the most commonly used method because it was developed before AM and has superior mechanical properties when compared to AM (7). However, AM has some advantages such as no material waste and no milling bur deterioration (12). In addition to the fabrication methods, the composition of denture base materials material has a role in the in results variations. The conventional and CAD-CAM milled are PMMA-Based while 3D printed either PMMA- or ester-based light polymerized resin (7, 11–13).

Although AM advantages, there are some drawbacks such as low mechanical properties and poor surface characteristics (14). The low physical and mechanical performance of AM has been attributed to the printing method (layer-by-layer) and polymerization method (photo-polymerization) (12, 14). Many attempts have been made to overcome these drawbacks by using different printing technologies, modifying printing parameters, and/or adding reinforcement and antimicrobial agents (15).

Studies have shown a relationship between *C. albican* adherence, colonization, and biofilm formation and the surface properties of denture base resins including roughness, porosities, and contact angle/hydrophilicity (1). Many studies (1, 16–18) compared surface properties of conventionally and CAD-CAM denture base resins in term of surface roughness and wettability and variation between findings was reported. Furthermore, surface roughness affected both hydrophobicity and adherence activities (13). While previous investigation demonstrated no linear relationship between the surface roughness of denture base resin and *C. albican* adhesion (19).

Authors of previous studies (1, 16–18) stated that the hydrophilic denture bases are less vulnerable to microbial adherence. CAD-CAM dentures base showed more wettability and showed reduced microbial adhesion compared with conventional one (1, 4, 5, 11). Another study (5) found that milled and 3D printed denture base materials were biocompatible and had similar surface characteristics. Fouda et al. (20), found that there was no difference in surface roughness between 3D printed, milled, and conventional rein, and that the adherence of *C. albicans* to all resins behaved similarly. Due the variations in surface properties of denture base resins and amount of microbial adhesions, authors suggested evaluating different CAD-CAM systems with different resins materials.

There have been no previous studies reviewing the microbiological antiadherence properties of digitally fabricated denture base resins. This review was conducted to evaluate the microbial adherence properties of CAD-CAM fabricated denture base resins in comparison to conventional ones, as well as to answer the research question “Does CAD-CAM milled and 3D printed denture base resins have microbiological antiadherence properties over to conventional ones?”

## 2 Materials and methods

### 2.1 Focused question

PICOS (Table 1) revealed the following study question; “Does CAD-CAM milled and 3D printed denture base resins have microbiological antiadherence properties over the conventional ones?”

**Table 1:** PICO model.

PICOS	
P: Participant	Denture base materials
I: Intervention	CAD/CAM (Milled and 3D printed) denture base resins
C: Comparison	Conventional heat-polymerized denture base
O: Outcome	Microbial adhesion

### 2.2 Study design

To conduct this review, the preferred reporting items for systematic reviews and meta-analysis (PRISMA) recommendations (21) were followed.

### 2.3 Search strategy

Searching for relative published literatures up to December 2023 was done through PubMed, Web of sciences, and Scopus databases. For the research strategy, both controlled and non-controlled descriptors and Boolean terms (OR, AND) were used (Table 2).

**Table 2:** Search strategy.

Databases	PubMed, scopus, and web of sciences
Keywords “Search combination”	[“Denture, Complete” (mesh) OR “Complete Denture” OR “Complete Dentures” OR “Dentures Complete”] AND (“Computer-Aided Design” [mesh] OR “Computer Aided Design” OR “Computer-Aided Designs” OR “Design, Computer-Aided” OR “Designs, Computer-Aided” OR “Computer-Assisted Design” OR “Computer Assisted Design” OR “Computer-Assisted Designs” OR “Design, Computer-Assisted” OR “Designs, Computer-Assisted” OR “Computer-Aided Manufacturing” OR “Computer Aided Manufacturing” OR “Manufacturing, Computer-Aided” OR “Computer-Assisted Manufacturing” OR “Computer Assisted Manufacturing” OR “Manufacturing, Computer-Assisted” OR “CAD-CAM”) AND (“3D printed” OR “additive manufacture” OR “RP Technologies” OR “Rapid Prototyping” OR “rapidly prototyped” OR “3D digital dentistry” OR “three-dimensional printing” OR “stereolithographic” OR “stereolithographically printed”) AND ((“Denture stomatitis” OR “Candida” OR “bioflm”) “Microbial adhesion” Antimicrobial agents; Antimicrobial efficacy, Candidiasis, Candida, Denture, Colonization, Stomatitis, *Candida albicans*)
Inclusion criteria	Full-text articles English language CAD-CAM denture base resins (milled and/or 3D printed) with or without comparison with Heat polymerized resin Microbial adhesion
Exclusion criteria	Other language rather than English Article didn't investigate microbial adhesion Only abstract Review articles, short communications, and case reports

### 2.4 Inclusion and exclusion criteria

In vitro studies, full article published in English language studies investigated microbial adhesions to CAD-CAM manufactured denture base resins (Milled and 3D printed) and compared with conventionally fabricated were targeted and included. Other studies that did not investigate CAD-CAM denture base resins and had no microbial adhesion test were excluded. In addition to the fallowing excluded studies: not published in English, case reports, reviews, short communications, letters to the editor, and only available in abstract form (Table 2).

### 2.5 Study selection, data extraction, and method of analysis

**Figure 1:**
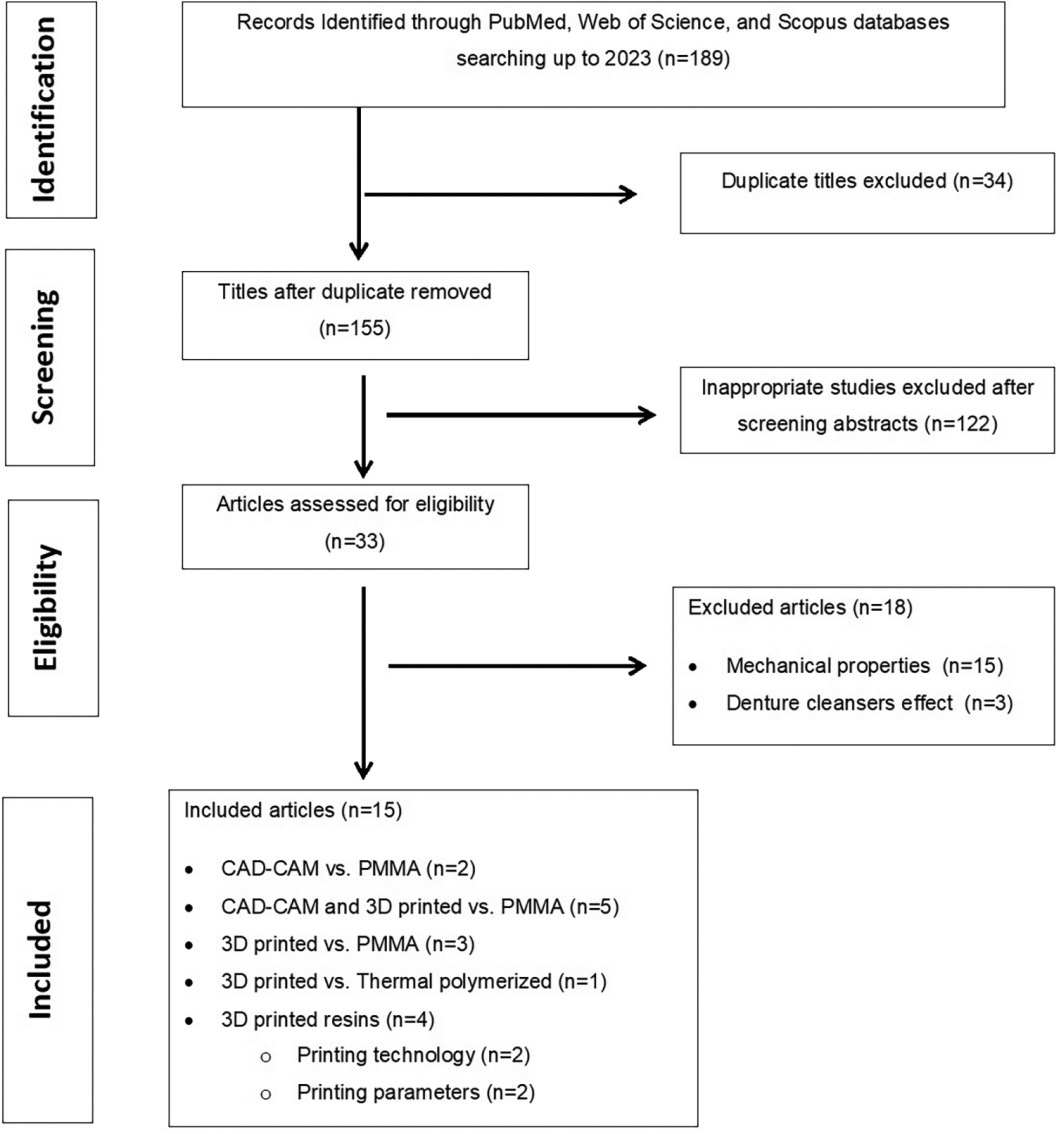
PRISMA flow chart of the study selection process.

Figure 1 shows how all articles were screened for included studies selections. Following the deletion of duplicated studies, the title and abstract of each study were individually screened and analyzed by two authors (F.K.A. and M.M.G) in accordance with the inclusion criteria. Disagreements are resolved through discussion between the two authors. Following approval, the full text of relevant studies meeting the eligibility criteria was read, followed by data collection and tabulation (Table 3). Data was descriptively assessed in terms of microbial adhesion to the milled and 3D printed materials, and then compared to conventional denture base resins.

**Table 3:** Included studies investigated microbial adhesion to CAD-CAM denture base resins.

Author/year/type of study/title	Denture base type/processing methods	Specimens dimensions/aging	Microbial species	Count methods and mean values	Modifications/variable	Outcome
Al-Fouzan et al., 2017 (22)	HP CAD/CAM Milling	Disc shape 3 × 10 mm	*C. albicans* NS	Colony-forming units (CFU)	Materials and fabrication methods	Less *C. albicans* adhesion to CAD/CAM fabricated denture bases compared with the conventional procedure.
Murat et al., 2019 (23)	HP CAD/CAM Milling	Disc shape 10 × 2 mm) TC (10,000 cycles )	*C. albicans* (ATCC 209)	Microscopic evaluation (Mean cell/field)	Uncoated and pellicle-coated specimens	Less *C. albicans* adhesion to CAD/CAM polymers when compared with conventional PMMA
Di Fiore et al., 2021 (24)	HP CAD-CAM milled 3D-printed	Disc shape 10 × 3 mm	*L. salivarius* (ATCC 33592) *S.mutans* (ATCC 25175) *C. albicans* (ATCC 18,804)	Colony-forming units (CFU)	Materials and fabrication methods Unpolished polished	CAD-CAM groups showed the lowest *C. albicans* adhesion with different incubation time.
Meirowitz et al., 2021 (25)	HP AP CAD-CAM milled 3D printing	Disc shape 12 × 2 mm	*C. albicans* (ES 58919)	Quantified using SEM	Materials and fabrication methods	CAD-CAM groups showed the lowest *C. albicans* adhesion followed by HP while 3D printing increased the microbial adhesion.
Shim et al., 2020 (26)	3D-printed	Disc shape 10 × 2 mm	*C. albicans* (KCCM 11282)	SEM observation	Printing orientation as a variable	Printing orientation showed a variations in *C. albicans* adhesion while 0-degrees showed the highest proportion followed by 45-degree then 90-degrees.
Li et al., 2022 (27)	AP 3D printing	Disc shape 10 × 2 mm	*C. albicans* NS	Relative metabolic activity	Printing technology and build angle	No significant between two AP and 3D printed denture in terms of *C. albicans’* adhesion. Additionally, printing technology and building angle did not influence *C. albicans* adhesion.
Totu et al., 2017 (28)	3D printing	NS	*C. scotti* NS	Dehydrogenase assays	0.2, 0.4, 0.6, 1, 2.5 by weight% of TiO_2_NP	TiO_2_ nanoparticles addition to 3D printed resins showed antibacterial effects, specifically on *Candida species*.
Jeon et al., 2022 (29)	3D-printing	Disc shape 15 × 5 mm	*C. albicans*, (ATCC 10231)	Colony forming unit (CFU/ml)	Modified with phytoncide oil A&B	Phytoncide A and B -modified 3D printed resin showed an optimum antifungal activities and improved surface properties with 6 wt% and 15 wt%.
Jeon et al., 2022b (30)	3D printing	Disc shape 15 ×5 mm	*C. albicans* (ATCC 10231)	Microscopically observed	Phytoncide oil type A	In terms of the antifungal activities, 3D printed resins containing micro-encapsulation of 6 wt% phytoncide oil showed the most adequate situations for clinical use.
Freitas et al., 2022 (31)	HP CAD-CAM Milled 3D printed	Disc shape 10 × 3 mm	*C. albicans* SN 425	Colony forming unit CFU/ml	NS	CAD-CAM milled showed low C*. albicans* adhesion while 3D printed reins showed more *C. albicans* adhesion.
Barros et al., 2023 (32)	Thermal-polymerized 3D printed	Rectangular 20 × 10 × 2 mm	*C. albicans* (ATCC 10231)	Colony forming units (CFU/ml); Staining with crystal violet followed by optical density reading	NS	Thermos-polymerized resin shoed more *C. albicans* when compared with 3D printed resin
Li et al., 2023 (33)	PMMA –ve 3D printed	Disc shape 10 × 2 mm	*C. albicans*	Cell counting kit-8 assay fluorescence microscopy optical density	Printing-layer thickness and build angle	Printing layer thickness affect *C. albicans* adhesion while orientations has no effect.
Koujan et al., 2023 (34)	PMMA CAD-CAM milled 3D-printed.	Rectangular 10 × 10 × 2 mm	*C. albicans* sc5314/ATCC MYA-2876	Colony-forming units (cfu/ml)	Fabrication method	Highest biofilm formation and adhesion was reported with 3D-printed resin in comparison to different resins.
Silva et al., 2023 (35)	PMMA 3D-printed	Disc-shaped 15 × 3 mm	*C. albicans* (SC5314)	Colony forming units count (CFU/mL), (ii) cellular metabolism (XTT assay), (iii) fluorescence and thickness of biofilm layers (confocal laser scanning microscopy)	Fabrication method	Denture base resin type affect *C. albicans* adhesion and colonization and 3D printed resins showed high tendency for *C. albicans* adhesion.
Osman et al., 2023 (36)	PMMA CAD-CAM milled 3D-printed	Curved part of the palatal of maxillary denture	*C. albicans* (ATCC 10231)	Field emission scanning electron microscopy (FESEM) XTT assay was used for the quantification	Fabrication method	3D-printing technology results in increased candida adhesion and the roughest surface topography of maxillary resin denture base as compared to conventional flask compression and CAD/CAM milling techniques

HP, heat polymerized acrylic resin; AP, auto polymerized acrylic resin; CAD-CAM, computer-aided-design-computer-aided-manufacture; PMMA, polymethylmethacrylate; NS, not stated.

### 2.6 Quality assessment

According to the method and criteria detailed in previous studies (37–39), the included studied were investigated for risk of bias (Table 4) for study quality assessment. Two independent authors screened included studies using the risk of bias tool guidelines (adapted and modified from Cochrane risk of bias tool) (37–39).

**Table 4:** Quality assessment and risk of bias considering aspects reported in material and methods section (risk of bias tool (adapted and modified from cochrane risk of bias tool).

Author/year	Allocation concealment	Sample size	Blinding	Assessment method	Selective outcome reporting	Risk of bias
Al-Fouzan et al., 2017 (22)	2	2	2	0	0	Moderate
Murat Et al., 2019 (23)	2	2	2	0	0	Moderate
Di Fiore et al., 2021 (24)	2	2	2	1	0	Moderate
Meirowitz et al., 2021 (25)	2	2	2	0	0	Moderate
Shim et al., 2020 (26)	2	2	2	0	0	Moderate
Li et al., 2022 (27)	2	0	1	0	0	Low
Totu et al., 2017 (28)	2	2	2	0	0	Moderate
Jeon et al., 2022 (29)	2	2	2	0	0	Moderate
Jeon et al., 2022b (30)	2	2	2	0	0	Moderate
Freitas et al., 2022 (31)	2	0	2	0	0	Moderate
Barros et al., 2023 (32)	2	2	2	0	0	Moderate
Li et al., 2023 (33)	2	0	1	0	0	Low
Koujan et al., 2023 (34)	2	2	2	0	0	Moderate
Silva et al., 2023 (35)	2	0	1	0	0	Low
Osman et al., 2023 (36)	2	0	2	0	0	Moderate

Score was calculated according to following criteria: clearly described (zero), insufficient or ambiguous (1), undisclosed a particular setting (2).

Calculating overall score per study and study quality as follow: Studies obtaining an overall score of 0–3 low risk (0–3), moderate risk (4–7), high risk (8–10) had.

## 3 Results

Out of 189, 15 studies (22–36) investigated the effect of microbial adhesion on CAD-CAM milled and 3D printed denture base resins. Two studies compared CAD-CAM milled resins to conventional denture base resins (22, 23), and 5 studies compared CAD-CAM milled and 3D printed resins with conventional denture base resins (24, 25, 31, 34, 36). Five studies investigated microbial adhesion to 3D printed resins (26–30); two studies investigated the effect of printing technology and printing orientation (26, 27), while three studies investigated 3D printed dentur base resins modified with TiO_2_ nanoparticles (28) and Phytoncide oil A&B (29, 30). *C. albicans* is most frequently investigated in all included studies except two studies included *Candida scotti* (28), and *Lactobacillus salivarius*, *Streptococcus mutans* (24). Different microbial assay methods were included; colony-forming units (CFU) (22, 24, 29, 31, 32, 34, 35), microscopic evaluation (Mean cell/field) (23, 25, 29), quantified using scanning electron microscopy (25, 26, 30, 33, 36), Relative metabolic activity (27), and Dehydrogenase assays (28).

Table 4 summarizes the quality assessment of the included studies. Out of the included studies, twelv studies revealed a moderate risk of bias, low risk was noted in three studies. Primarily the risk was attributed to the lack of allocation concealment, sample size calculation and examiner blinding.

Despite differences in denture base resin type and microbial assay between the included studies, CAD-CAM milled denture base resins demonstrated the lowest microbial adhesion compared to conventional, while 3D printed dentures demonstrated the highest microbial adhesion (31). For 3D printed resins, the proportions of microbial adhesion were highest at 0 degrees and lowest at 90 degrees (25). While the combined effect of printing technology (SLA and DLP) and printing orientation had no effect on microbial adherence (27). On the level of 3D printed resin modifications, both additives TiO_2_ nanoparticles (28), and Phytoncide oil A&B (29, 30) decreased the microbial adhesion.

## 4 Discussion

In subtractive method, the fabrication of denture base from prefabricated PMMA discs improved the mechanical behavior as well as the surface properties when compared to conventional heat polymerized denture base (4). As a result of the good surface properties of CAD-CAM milled denture base resins, less microbial adherence is expected. This was confirmed by all authors (22–36), who reported that milled denture base resins had lower *C. albicans* adhesion f and reduce the occurrence of DS in long-term denture use. Di Fiore et al. (24) used scanning electron microscope (SEM) to examine the surface topography of each material and found that the conventional ones had multiple grooves and deep scratches with a porous surface, whereas CAD-CAM milled had a smooth surface with fewer scratches.

3D printed resins have low surface properties when compared to milled and conventional ones. In between the included studies, two studies (24, 25) compared the *C. albicans* adhesion of 3D printed with CAD-CAM milled denture base resins and conventional and found that 3D printed resins exhibited significantly more microbial adhesion. This was primarily due to the nature of the printing technology; layer-by-layer object building and this layering technique resulted in stepwise edges on the specimens' surfaces (24, 25). Based on SEM analysis of specimens' surface by Di Fiore et al. (24), 3D printed resins showed more surface irregularities, multiple dots, and serrations that probably attributed to the layering of printed objects and the polymerization method (24). Previous researches (4, 31) assessed the surface roughness of 3D printed resins and found rougher surfaces than conventional even when the printing parameters were changed. While another study found no difference between CAD-CAM milled and 3D printed denture base resins in terms of surface roughness (5, 31).

The printing technology was thought to be a factor influencing the properties of 3D printed objects (15). SLA and DLP are the most commonly used technologies for fabricating denture bases (40). Surface roughness differences were reported between the two technologies (27), with SLA exhibiting irregular surfaces and DLP printed specimens exhibiting clear and regular texture. However, Li et al. found no difference in *C. albicans* adhesion between SLA and DLP technology (27).

Another factor was the printing orientation (26, 27), which could result in different surface patterns depending on the printing layer directions (27). According to Li et al. (33), printing orientation has a significant impact on Ra values regardless of printing technology (25, 41). Some surface features were observed with different printing angles (45° and 90°) and exhibited a ladder-like surface structure (33). Roughness changes in relation to building direction were caused by the height of step edges and the stepwise connection between printed layers (26). Li et al. (33) investigated the same orientations and two AM methods (SLA and DLP) and found no significant differences in *C. albicans* adhesion. When the printing orientation and post-curing time were varied, Al-Dulaijan et al. found no change in the surface roughness of 3D printed resins (42). With changing printing orientation, the layer direction is changed and affected the specimens' surface, 0-degree is expected to be smooth as the surface of specimens formed by the last printed layer (26). However, Shim et al. (26), printed specimens with different orientations (0-, 45-, and 90-degree) and evaluated the microbial adhesion and found that 0-degree showed the highest proportion. This conflict (smooth surface with more *Candida* adherence) could be clarified based on the surface wettability of 0-egree showed the highest hydrophilicity value according to Shim et al. (26). These findings support the hypothesis that the microbial adhesion of 3D printed resins is primarily due to surface features and wettability (1). Surface coatings of conventional PMMA denture base resins were suggested as a possible method to create a smooth surface denture base to overcome the low surface properties (43). However, this has not been investigated as of yet, so further research is advised.

Incorporating antifungal agents within the 3D printed fluid resin was another method for improving antimicrobial activity (28, 30). Two antimicrobial agents, TiO_2_ nanoparticles (28) and Phytoncide oil A&B (29, 30), were successfully added as antimicrobial agents to 3D printed resins. TiO_2_'s antimicrobial effect is primarily due to its photocatalytic effect, in which UV irradiation results in oxidization decomposition (44, 45). By coordinating electron-donating groups, this effect resulted in the deactivation of cellular enzymes. This process ended by gabs in cell allowing higher permeability cell death (44). As phytoncide concentrations increased, the viability of fungal cells and optical density decreased, consequently increasing the number of atypical cells morphologically (45). In addition to having antimicrobial effect, phytoncide-filled microcapsules; the microbial adhesion, attachment, and growth were inhibited significantly when incorporated into 3D printed resins regardless of pH value (29, 30). The effect of phytoncide-filled microcapsule concentrations was found to significantly reduce *C. albicans* adhesion with increasing concentrations. In addition, the surface roughness increased with concentration but had no effect on *C. albicans* adhesion, confirming the antifungal activity of 3D-printed resin containing phytoncide-filled microcapsules (29, 30). As a result of the antifungal activities being reported and demonstrating significantly less candida adhesion when compared to the unmodified one. Both studies (29, 30) recommended using the introduced modified-3D printed resins for denture base fabrication.

Although modified 3D printed resins were found to have a positive antifungal effect, the lack of comparison with conventional or CAD-CAM milled denture base resins was considered a limitation in both studies (not used as control). In light of the findings of both studies, additional research on antimicrobial-modified 3D printed resins in comparison with conventional and CAD-CAM denture base resins is recommended rather than a comparison with the unmodified one. This was due to the fact that the modification effect was good, but still highly significant when compared to the conventional method. Moreover, microbial adhesion and related surface properties testing in terms of hydrophobicity are required (1).

Based on the review findings, CAD-CAM milled denture base resins were found to be the most appropriate materials for denture base fabrication with low microbial adhesion. 3D printed resins were more susceptible to microbial adhesion and require additional research with different printing technologies, resin modifications, or printed object surface modifications before clinical recommendations.

Although the importance of the subject in which this systematic review was able to compare the most recent literatures on microbial adhesion to different denture base resins, nevertheless, the included articles were limited to *in vitro* studies reducing the scientific evidence of study point. In addition to other limitations due to the small number of studies included, as well as differences in resin type, fabrication method, variables investigated, and microbial assessment methods. All of these constraints made it difficult to reach a clear conclusion based on the study objective. As a result, a future systematic review was suggested.

## 5 Conclusions

CAD-CAM milled denture base resin had lower microbial adhesion. When compared to conventional heat polymerized and CAD/CAM milled denture base resins, 3D printed resins have a high tendency for microbial adhesion due to their poor surface properties. The addition of antimicrobial agents to 3D printed resins reduced microbial adhesion. However, more research is needed to prove the effects of these additives when combined with different printing parameters.

## Data Availability

The original contributions presented in the study are included in the article/Supplementary Material, further inquiries can be directed to the corresponding author.
